# Two glutamic acid residues in the DNA-binding domain are engaged in the release of STAT1 dimers from DNA

**DOI:** 10.1186/1471-2121-13-22

**Published:** 2012-08-24

**Authors:** Verena Koch, Julia Staab, Volker Ruppert, Thomas Meyer

**Affiliations:** 1 Klinik für Kardiologie, Philipps-Universität Marburg, Marburg, Germany; 2 Klinik für Psychosomatische Medizin und Psychotherapie, Georg-August-Universität Göttingen, Waldweg 33, 37073, Göttingen, Germany

## Abstract

**Background:**

In interferon-γ-stimulated cells, the dimeric transcription factor STAT1 (*s*ignal *t*ransducer and *a*ctivator of *t*ranscription 1) recognizes semi-palindromic motifs in the promoter regions of cytokine-driven target genes termed GAS (*g*amma-*a*ctivated *s*ites). However, the molecular steps that facilitate GAS binding and the subsequent liberation of STAT1 homodimers from these promoter elements are not well understood.

**Results:**

Using a mutational approach, we identified two critical glutamyl residues within the DNA-binding domain adjacent to the phosphodiester backbone of DNA which efficiently release phospho-STAT1 from DNA. The release of STAT1 dimers from DNA enhances transcriptional activity on both interferon-driven reporter and endogenous target genes. A substitution of either of the two glutamic acid residues broadens the repertoire of putative binding sites on DNA and enhances binding affinity to GAS sites. However, despite elevated levels of tyrosine phosphorylation and a prolonged nuclear accumulation period, the STAT1 DNA-binding mutants show a significantly reduced transcriptional activity upon stimulation of cells with interferon-γ. This reduced transcriptional response may be explained by the deposition of oligomerized STAT1 molecules outside GAS sites.

**Conclusions:**

Thus, two negatively charged amino acid residues in the DNA-binding domain are engaged in the liberation of STAT1 from DNA, resulting in a high dissociation rate from non-GAS sites as a key feature of STAT1 signal transduction, which positively regulates cytokine-dependent gene expression probably by preventing retention at transcriptionally inert sites.

## Background

A variety of different transcription factors are involved in the execution of genetic programs, and sequence-specific DNA binding is a hallmark of transcriptional regulation. Central to their role as gene-specific transcription factors is their ability to recognize distinct elements in the promoter regions of responsive genes. Typically, transcription factors bind with high affinity and specificity to short motifs of DNA via protein surfaces that are complementary to a particular base sequence. Although there is often some flexibility in the nucleotide sequence that is recognized, certain key bases are crucial for interactions with the DNA-binding domain. The STAT (*s*ignal *t*ransducer and *a*ctivator of *t*ranscription) proteins constitute a paradigmatic family of evolutionary conserved transcription factors with a modular domain arrangement [1,2]. In mammals, seven different members of the STAT family are expressed, all of which consist of three proteolytically separable structural subunits. An amino-terminal domain is separated by a protease-sensitive linker peptide from a large core structure containing the central DNA-binding domain and a carboxy-terminal transactivation domain [3-6]. The amino-terminal domain of about 130 residues is folded into a unique hook-shaped architecture that facilitates cooperative DNA binding through the formation of tetramers [7-10]. The large core domain encompasses several distinct functional domains beginning at the amino-terminal end with a coiled-coil domain, which is engaged in protein-protein interactions [11]. The DNA-binding domain displays an immunoglobulin fold and is required for the binding of tyrosine-phosphorylated STAT dimers to semi-palindromic DNA sequences termed *g*amma-*a*ctivated *s*ites (GAS) [12]. The neighboring linker region consists of a unique all-alpha helical structure and assists in binding to GAS elements [13]. The Src homology 2 (SH2) domain mediates recruitment to phospho-tyrosyl residues in the intracellular receptor tails and the formation of tyrosine-phosphorylated STAT dimers [14,15]. The carboxy terminus containing the transactivation domain is most variable in size and sequence among the different STAT family members and is frequently subjected to alternative splicing [16].

The STATs are best known for their function as cytokine-activated transcription factors, which play a vital role in such diverse processes as growth control, proliferation, and immune activation [17,18]. The triggering of cytokine receptors initiates a cascade of phosphorylation steps that causes auto-phosphorylation of non-covalently attached JAK kinases. The activated JAK kinases then phosphorylate signature tyrosine residues in the intracellular receptor tails, thereby, creating phospho-tyrosine docking sites for the STAT SH2 domain [19]. Phosphorylation of a single tyrosine residue in the STAT carboxy terminus results in a structural change within the STAT dimer that shifts from an “antiparallel” to a DNA-bound “parallel” conformation [20-22]. Tyrosine-phosphorylated STAT enters the nucleus via importin-α/β-mediated transport [23-27] and binds to semi-palindromic GAS elements in the promoter region of cytokine-responsive genes that contain the consensus sequence 5^′^-TTC(N)_3-4_GAA-^′^3 [3,28,29]. STAT proteins are then dephosphorylated by nuclear tyrosine phosphatases, some of which have been identified, such as the Tc45 phosphatase for inactivation of STAT1 [30-32]. Additionally, unphosphorylated STAT1 molecules translocate constitutively between the cytoplasm and the nucleus in both directions through direct contacts with nucleoporins located in the nuclear pore complex [33-36].

In contrast to this high-affinity GAS binding, much less is known about the molecular processes that ensure the release of STAT1 dimers from DNA. In the following, we report on a novel and simple mechanism that allows STAT1 homodimers to disengage from DNA. Additionally, we show that a high dissociation rate from non-specific DNA and a preserved sequence-specific discrimination between GAS and non-GAS sites are both required for optimal transcriptional activation. Moreover, we directly confirm that DNA-bound STAT1 molecules are protected from dephosphorylation *in vivo*, pointing to the essential role of non-specific DNA binding in the search for cytokine-regulated promoter elements.

## Results

### Mutation of two glutamyl residues in the DNA-binding domain results in increased tyrosine phosphorylation of STAT1

**Figure 1:**
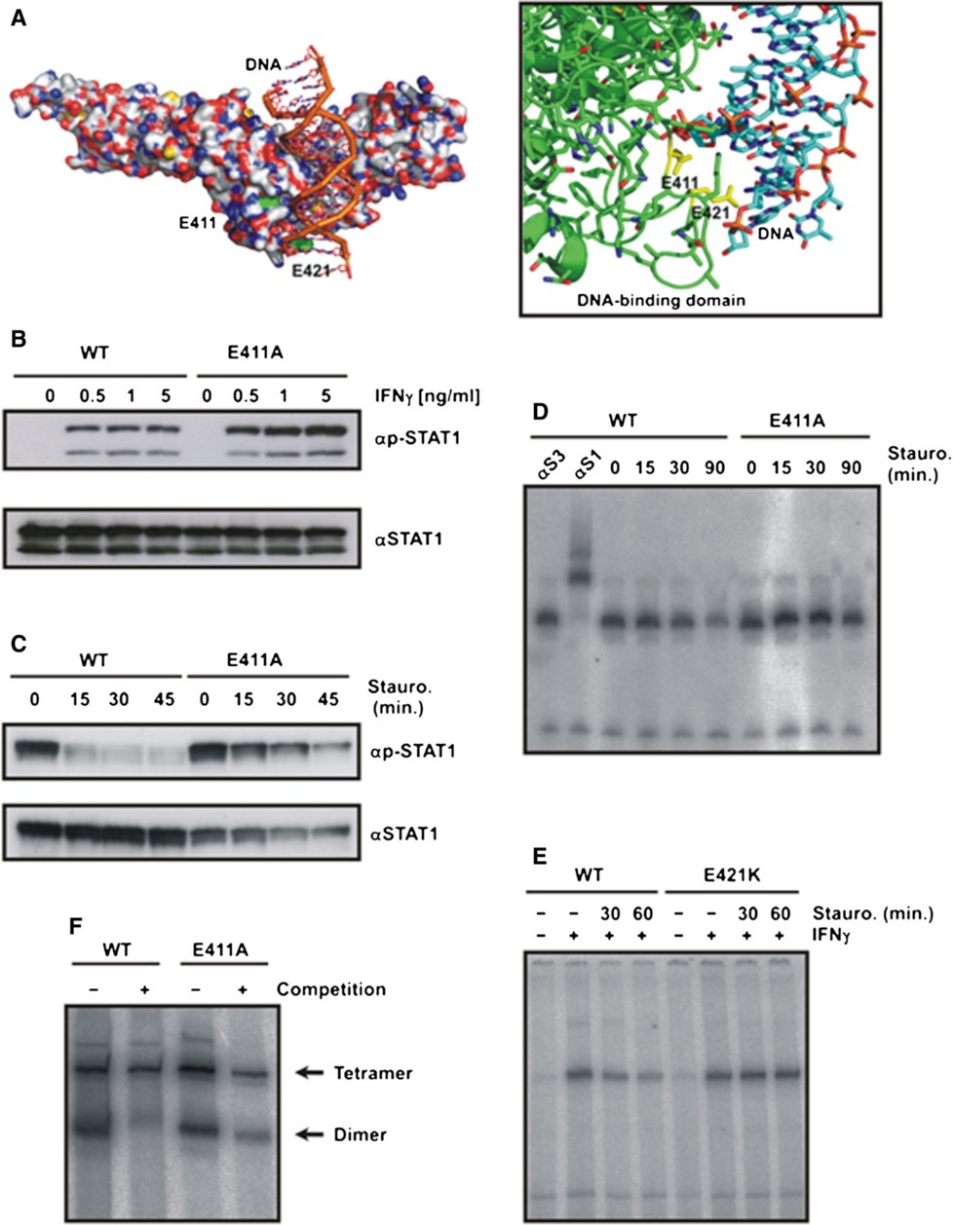
**Substitution of two residues in the DNA-binding domain of STAT1 transcription factor leads to increased tyrosine phosphorylation and prolonged GAS binding.** (**A**) Localization of critical glutamyl residues 411 and 421 in the DNA-binding domain adjacent to the DNA molecule. Depicted is a part of the crystal structure of truncated STAT1 bound to DNA [3] with the side chains of E411 and E421 pointing towards the backbone of DNA (on the left; molecular surface, on the right; detail from ribbon diagram with glutamyl residues marked in yellow). (**B**) Hyperphosphorylation of STAT1-E411A upon stimulation of cells with increasing concentrations of IFNγ. Equal cell numbers of HeLa cells expressing either wild-type or mutant STAT1, both tagged with green fluorescent protein (GFP), were exposed to increasing concentrations of IFNγ for 45 min before tyrosine phosphorylation was tested for in cell lysates. A representative result from a Western blot experiment with a STAT1-specific phospho-tyrosine antibody (top panel) and the corresponding re-blot after the stripping off of bound immunoreactivity and re-incubation with pan-STAT1 antibody C-24 (bottom panel) is shown. The upper band on each blot marks recombinant GFP-tagged STAT1, whereas the lower band corresponds to native STAT1. (**C**) Decreased tyrosine dephosphorylation of STAT1-E411A upon inhibition of kinase activity. Equal numbers of U3A cells expressing wild-type or mutant STAT1 were prestimulated for 45 min with 5 ng/ml IFNγ and subsequently exposed to 500 nM of the kinase blocker staurosporine for the durations indicated. Note that staurosporine treatment resulted in the rapid loss of tyrosine-phosphorylated STAT1-WT, whereas the mutant was partially resistant to the inactivating staurosporine effect. (**D**) Prolonged GAS binding of STAT1-E411A following treatment of IFNγ−prestimulated cells with staurosporine. U3A cells expressing either wild-type or mutant STAT1 were stimulated for 45 min with IFNγ and thereafter the medium was replaced and the cells treated with 500 nM staurosporine. Cell lysates were equilibrated with radioactively labeled DNA containing a high-affinity STAT binding site (GAS) before being loaded onto a gel. A representative gel-shift including two lanes with supershifts using either anti-STAT3 (αS3) or anti-STAT1 (αS1) antibody, respectively, is shown. (**E**) STAT1-E421K resists dephosphorylation as determined by gelshift assay using extracts from IFNγ−prestimulated U3A cells that have been exposed to staurosporine for the indicated times. (**F**) Tetramer stabilization of the E411A mutant. Extracts from IFNγ−stimulated U3A cells expressing either wild-type or mutant STAT1 were incubated *in vitro* with [^32^P]-labeled DNA containing a tandem GAS site. The reactions were either left unchallenged (−) or challenged for 30 min with a 750-fold molar excess of a single, unlabeled GAS site (+ competition). At the margin of the EMSA gel the positions of tetrameric (top arrow) and dimeric (bottom arrow) STAT1 are marked.

In an effort to identify DNA-binding mutants of STAT1 with preserved GAS recognition, we performed a mutational study on the STAT1 molecule and generated numerous point mutants in the DNA-binding domain. A critical glutamic acid residue at position 411 in the full-length protein was found to be conserved in STAT1, STAT2, STAT3 and STAT4 of the human STAT family. Structural data of the DNA-bound STAT1 dimer revealed that the carboxyl group of E411 has a distance of 5.7 Å from the phosphodiester backbone of the co-crystallized DNA double helix and that there is no other residue in the STAT1 molecule to prevent its free access to DNA (Figure 1A). This exposed residue on the surface of the DNA-binding domain was mutated to alanine and the corresponding mutant was expressed in HeLa and STAT1-negative U3A cells by transfection with pSTAT1-GFP. STAT1-E411A was normally expressed and no indication of structural instability was detected neither by Western blotting (Figure 1B,C) nor gelshift experiments (Figure 1D). In response to stimulation of cells with interferon−γ, the E411A mutant was tyrosine-phosphorylated (Figure 1B,C) and bound to a single optimal GAS site in the M67 probe similar to the wild-type protein (Figure 1D).

We then performed kinetic studies on tyrosine dephos-phorylation in IFNγ−prestimulated U3A cells which were subsequently exposed to 500 nM of the potent ATP-competitive kinase blocker staurosporine [37]. Treatment with the kinase inhibitor resulted in a rapid and complete dephosphorylation of wild-type STAT1 within 15 min, while the E411A mutant exhibited a much lower dephosphorylation rate (Figure 1C). Moreover, the ratio of tyrosine-phosphorylated STAT1 to the total intracellular STAT1 pool, which also contained unphosphorylated protein, was elevated in the mutant as compared to its wild-type counterpart (Figure 1B,C). Similarly, mutation of another glutamic acid residue in position 421, which also points with its side chain in the direction of the DNA double helix (Figure 1A), resulted in defective dephosphorylation and increased DNA-binding activity (Figure 1E).

When we tested for cooperative DNA binding resulting from the ability to form stable tetramers on tandem GAS sites by means of EMSA analysis, we found no significant difference between the wild-type and mutant STAT1 (Figure 1F). Both variants bound independently to either GAS site, resulting in both fast migrating STAT1/DNA complexes containing a single STAT1 dimer and slow migrating complexes with two dimers. When such complexes were challenged with a 750-fold molar excess of unlabeled M67 duplex oligonucleotides, the tetrameric complexes resisted displacement due to stable tetramerization. In contrast, the dimeric complexes of both wild-type and mutant STAT1 were either totally or partially displaced, indicative of cooperative DNA binding. Thus, substitution of either of the two conserved glutamyl residues in position 411 or 421 of the full-length STAT1 molecule critically impaired the continuous dephosphorylation/rephosphorylation cycle and resulted in elevated and prolonged tyrosine phosphorylation levels. However, binding to an optimal GAS site as well as cooperative DNA binding due to tetramer stabilization was unaltered.

### Tyrosine-phosphorylated STAT1-E411A protects co-expressed endogenous STAT1 from inactivation

**Figure 2:**
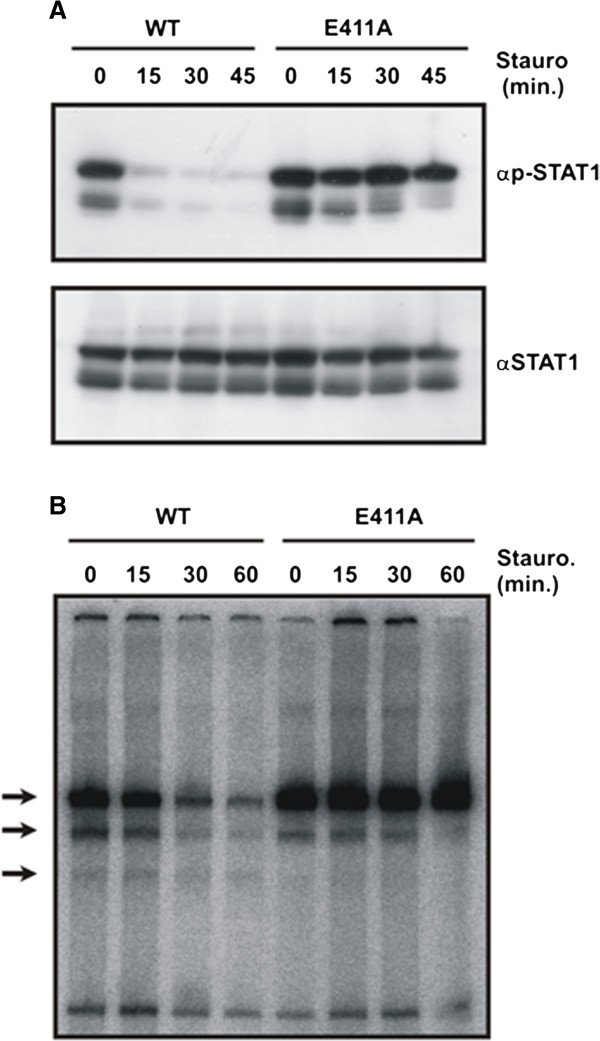
**Co-expressed endogenous STAT1 is protected from inactivation through heterodimer formation with STAT1-E411A.** (**A**) Kinetics of STAT1 dephosphorylation in HeLa cells expressing green fluorescent protein-tagged wild-type or mutant STAT1. Cells were stimulated with 5 ng/ml IFNγ and subsequently exposed to 500 nM staurosporine for the indicated times. The phosphorylation level was assessed by means of a Western blot using a phospho-tyrosine-specific STAT1 antibody (top panel). The membrane was then stripped and re-incubated with a pan-STAT1 antibody (bottom panel). (**B**) The E411 mutant resists the inhibitory effects of staurosporine and reacts with co-expressed native STAT1 via heterodimerization. HeLa cells expressing either STAT1-WT-GFP or E411A-GFP were stimulated for 45 min with IFNγ before staurosporine was added for the durations indicated. Whole cell lysates were prepared and incubated *in vitro* with [^32^P]-labeled M67 DNA containing a high-affinity STAT binding site. Resulting STAT1-DNA complexes were separated using an electrophoretic gel shift assay. Note the formation of STAT1-GFP homodimers (top arrow), heterodimers of GFP-tagged and native STAT1 (middle arrow), as well as homodimers of native STAT1 (bottom arrow).

The partial insensitivity of STAT1-E411A towards the inhibitory effect of staurosporine was independent of the cell type, as prolonged tyrosine phosphorylation was also detected in HeLa cells (Figure 2A). Similar to the effect in U3A cells, stimulation with an equal concentration of IFNγ resulted in higher levels of tyrosine-phosphorylated mutant STAT1 as compared to the wild-type. Also in cytokine-stimulated HeLa cells, the ratio of tyrosine-phosphorylated STAT1 to the total STAT1 was increased, indicating that hyperphosphorylation reflects an inherent property of the mutant. In line with the altered kinetics of tyrosine phosphorylation, we found that, also in HeLa cells, DNA-binding activity to the M67 site was enhanced following 45 min of stimulation with IFNγ (Figure 2B). Moreover, in the presence of staurosporine the rate of dephosphorylation was decreased in the point mutant as compared to the wild-type, thus confirming that the mutant E411A displayed a prolonged state of DNA binding (Figure 2B).

Interferon-prestimulated HeLa cells expressing endogenous STAT1, in addition to either the GFP fusion of wild-type STAT1 or its GFP-tagged mutant, were subjected to the inhibitory effect of staurosporine. In cells expressing STAT1-E411A-GFP, not only did the mutant phospho-protein resist staurosporine treatment much better, endogenous STAT1 was also partially insensitive, as revealed by its prolonged tyrosine phosphorylation (Figure 2A) and enhanced DNA-binding activity (Figure 2B). Thus, the presence of the E411A substitution protects also co-expressed native STAT1 protein from its rapid inactivation. This finding suggested that the mutant STAT1 protein interacts with endogenous STAT1 in a way that impairs access to the inactivating nuclear phosphatase.

### Diminished nuclear export of tyrosine-phosphorylated STAT1-E411A

**Figure 3:**
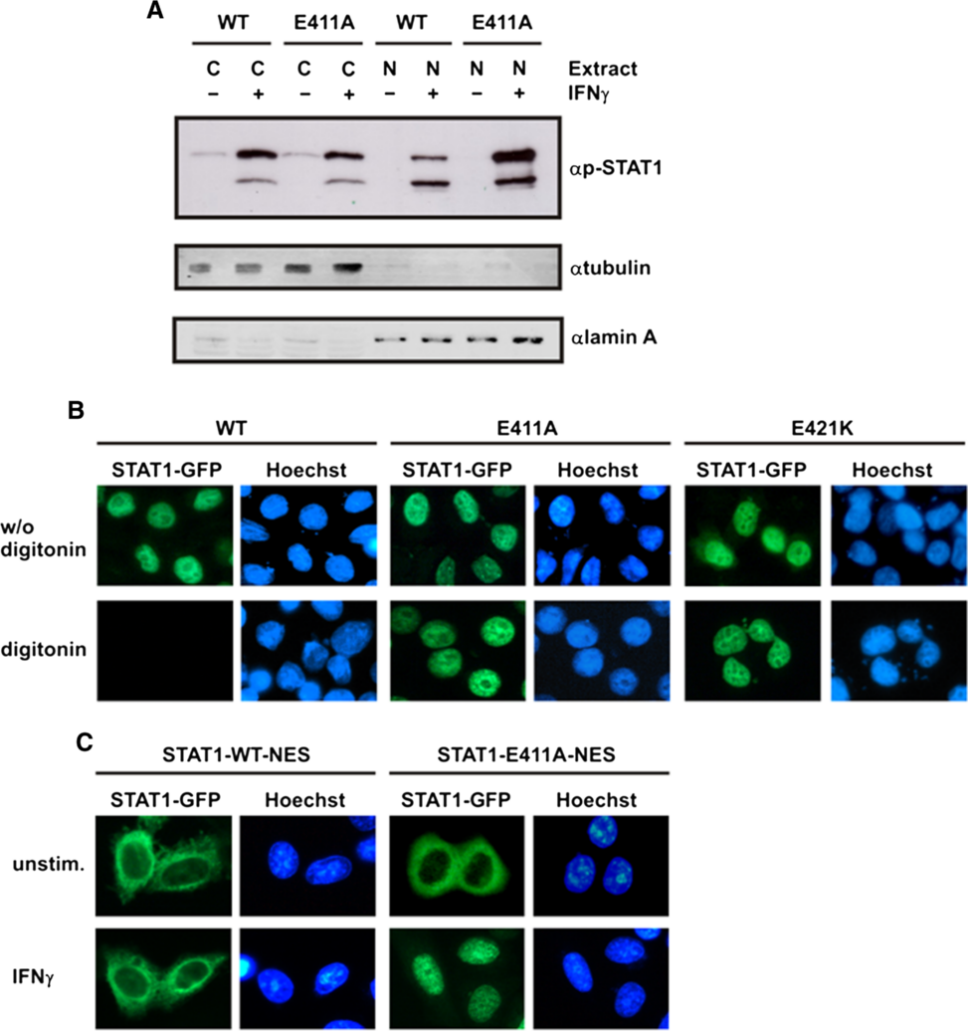
**Mutation of E411 results in diminished nuclear export of STAT1.** (**A**) Altered nucleocytoplasmic distribution of the E411A mutant. HeLa cells expressing GFP-tagged STAT1 variants were either unstimulated (−) or stimulated with IFNγ (+), as indicated. The levels of phosphorylated STAT1 were probed separately in cytosolic and nuclear extracts by means of Western blotting using a phospho-tyrosine-specific STAT1 antibody. Similar amounts of endogenous phospho-STAT1 in both transfections were loaded onto the gel (lower band). The concentration of phosphorylated STAT1-E411A-GFP (upper band) in nuclear extracts (N) exceeded that of the wild-type, whereas, conversely, in cytosolic extracts (C) there were lower amounts as compared to the wild-type protein. The purity of cytoplasmic/nuclear extracts was assessed by simultaneous incubation of the blot membrane with anti-ß-tubulin and anti-lamin A antibodies followed by secondary species-specific IRDye 680LT and 800CW antibodies. (**B**) Reduced nuclear export kinetics of STAT1-E411A and -E421K. HeLa cells expressing fusions of green fluorescent protein with either wild-type or mutant STAT1 were prestimulated for 45 min with IFNγ to induce nuclear accumulation (top panel) and then treated for 6 min in the presence of 50 μg/ml digitonin in transport buffer (bottom panel). Fluorescence micrographs of formaldehyde-fixed cells are shown, demonstrating the amount of nuclear STAT1-GFP and the localization of the corresponding Hoechst-stained nuclei. (**C**) The E411A mutation restores defective nuclear accumulation of the STAT1-NES-GFP construct. HeLa cells were transfected with pSTAT1-NES-GFP, which coded for a transferable nuclear export signal (NES) situated between the cDNAs for full-length STAT1 and GFP. Cells expressing wild-type STAT1-NES-GFP or the respective E411A variant thereof were either left untreated or stimulated with IFNγ.

We then tested whether the nucleocytoplasmic distribution differed between wild-type and the mutant (Figure 3A). Cytosolic and nuclear extracts were prepared from either unstimulated or IFNγ−stimulated HeLa cells expressing STAT1-GFP fusion proteins and the levels of tyrosine phosphorylation were subsequently probed by means of Western blotting. It was found that, in nuclear extracts, the amount of phospho-STAT1 was significantly higher for mutant STAT1 as compared to the wild-type, and vice versa, in cytosolic extracts there was slightly more phosphorylated wild-type protein. Thus, the concentration of phospho-STAT1 in the nucleus was higher when the critical glutamyl residue was displaced by alanine, resulting in a more pronounced nuclear retention. Again, the amount of endogenous phospho-STAT1 was higher in HeLa cells expressing the E411A mutant as compared to its wild-type GFP fusion.

To confirm the altered nucleocytoplasmic shuttling properties of the mutants by a different approach, we performed a permeabilized cell transport assay [38] (Figure 3B). HeLa cells expressing GFP-tagged wild-type STAT1 or the respective glutamyl mutants were stimulated for 45 min with IFNγ to induce nuclear accumulation of the recombinant fusion proteins. Subsequently, the cells were either immediately fixed or incubated for 6 min with 50 μg/ml digitonin on ice before fixation. Treatment with digitonin at this concentration selectively permeabilized the plasma membrane, thereby, releasing cytoplasmic proteins, while the integrity of the nuclear envelope remained intact. As expected, stimulation with IFNγ resulted in the nuclear accumulation of all GFP-tagged STAT1 variants (Figure 3B, top panel). However, permeabilization by digitonin completely abrogated the pre-existing nuclear presence of STAT1-WT-GFP, while the two mutants remained accumulated in the nucleus (Figure 3B, bottom panel). Thus, the nuclear export rate of the mutants was critically reduced as compared to the wild-type protein.

By adding a transferable nuclear export signal (NES) to GFP-tagged STAT1 [39], we have gathered further evidence for an altered DNA binding of the mutants. In resting cells, STAT1-NES-GFP showed a cytoplasmic redistribution as compared to the nearly pancellular localization of STAT1-GFP, which resulted from enhanced nuclear export (Figure 3C). Also in contrast to STAT1-WT, the NES adduct failed to accumulate in the nuclei of interferon-stimulated cells, because the enhanced nuclear export rate competed with nuclear retention on DNA. Interestingly, however, nuclear accumulation was fully restored in the additional presence of the E411A mutation. This observation clearly confirms that high-affinity DNA-binding is the underlying phenotype of the E411A mutant.

### The mutant E411A exhibits high-affinity GAS binding and has a broad repertoire of non-optimal binding sites

**Figure 4:**
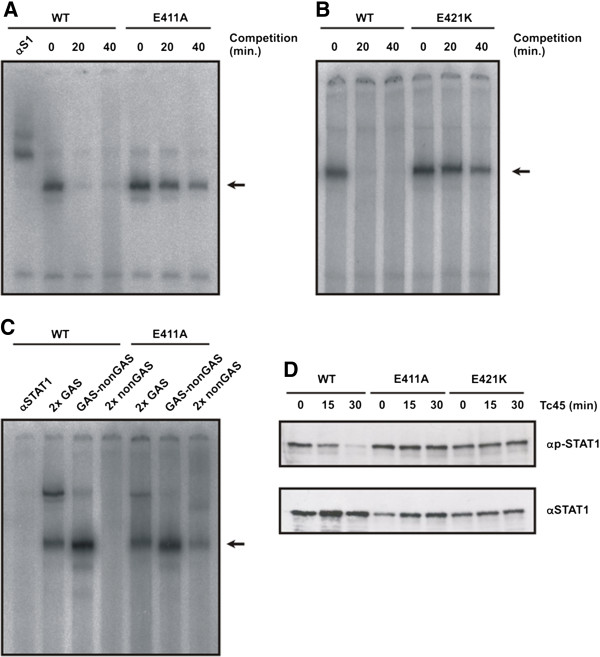
**Replacement of glutamyl residues in position 411 or 421 results in STAT1 mutants with a decreased dissociation rate from DNA and high-affinity binding to non-canonical binding sites.** (**A**) Comparison of the dissociation rates between STAT1-WT and STAT1-E411A on a DNA fragment containing a single STAT binding site (M67). Whole cell extracts from U3A cells were incubated with radioactively labeled DNA for 15 min and, subsequently, a 750-fold molar excess of unlabeled DNA was added for the durations indicated before the samples were loaded onto a polyacrylamide gel. In the first lane, anti-STAT1 antibody C-24 was present in the EMSA reaction for identification of STAT1-DNA complexes which are marked with an arrow. (**B**) Similar experiment as in (**A**), except that U3A cells expressing E421K were used. (**C**) Sequence requirements for binding of mutant STAT1 to DNA. Whole cell extracts from U3A cells expressing either STAT1-WT or the E411 mutant were incubated with various [^32^P]-labeled DNA molecules containing two single consensus GAS sites (2x GAS), a consensus and mutant GAS site in tandem arrangement (GAS-nonGAS) or two non-GAS sites (2x non-GAS). Lane 1 is similar to lane 2 except that the reaction additionally contained STAT1-specific antibody C-24 (αSTAT1). Note that wild-type STAT1 did not bind to 2x non-GAS, while the same DNA fragment exhibits a weak affinity for STAT1-E411A. The dimeric STAT1-DNA complexes are marked with an arrow. (**D**) An *in vitro* dephosphorylation assay shows that DNA-bound E411A and E421K are protected from Tc45-catalyzed inactivation. Whole cell extracts from reconstituted U3A cells were incubated with the recombinant Tc45 phosphatase for the indicated times and the reactions were then subsequently probed for phospho-STAT1 levels by means of Western blotting (αp-STAT1). The membrane was stripped and re-incubated with the pan-STAT1 antibody C-24 (αSTAT1).

We now performed experiments that were aimed at elucidating the molecular basis behind the altered activation/inactivation cycle of the two STAT1 glutamyl mutants. Putative mechanisms for hyperphosphorylation of STAT1 variants include diminished nuclear import due to mutations in either the dimer-specific nuclear import signal or other regions of the STAT1 molecule, which interact with importin-α, as well as altered binding kinetics to DNA. STAT1 mutants with impaired nuclear import are exposed to the high kinase activity and comparably low phosphatase activity in the cytosol [33], and a DNA-binding mutant termed STAT1-dna^plus^ has been described, which failed to recognize GAS probes in gelshift assays [40]. We found that the glutamyl mutants do not fall into either of these categories, since, upon cytokine stimulation of cells, the mutants were imported normally into the nucleus (Figure 3A,B), thus ruling out defective nuclear accumulation as the cause for their hyperphosphorylation. Furthermore, the mutants recognized GAS elements in mobility shift assays, clearly distinguishing them from STAT1-dna^plus^, in which three other residues in the DNA-binding domain were substituted for positively charged residues (Thr327Arg, Val426His, Thr427His). The altered DNA-binding kinetics of the glutamyl mutants was evident in competition experiments employing challenge with excess unlabeled GAS oligonucleotides (Figure 4A,B). These experiments clearly revealed that a dramatically reduced dissociation rate from DNA constitutes their underlying phenotype. In the mutants, the release from optimal DNA-binding sites was critically impaired, resulting in a longer half-life of GAS-bound dimers as compared to wild-type STAT1. Thus, the stability of preformed protein-DNA complexes differed significantly between the two mutant STAT1 proteins and their wild-type counterpart.

In order to compare the sequence requirement for DNA binding between the E411A mutant and wild-type STAT1, we used non-optimal GAS elements as molecular probes in mobility shift assays (Figure 4C). Both the wild-type and the mutant bound with high affinity to oligonucleotides containing a single GAS site. However, STAT1-E411A also reacted with a mutated probe which, due to the exchange of two base pairs, contained no consensus GAS element. Although binding to this 2x nonGAS probe was weaker than to either GAS-nonGAS or tandem GAS oligos, there was a detectable formation of DNA-bound STAT1 dimers not requiring an intact GAS site for DNA binding. Thus, in the presence of excess unlabeled GAS oligos, the E411A mutant bound to DNA not only with a higher affinity than the wild-type molecule, but also showed a relaxed sequence requirement for interaction with DNA.

*In vitro* dephosphorylation assays, using whole cell extracts from reconstituted U3A cells in the presence of the STAT1-inactivating Tc45 phosphatase, confirmed that the two glutamyl mutants are indeed DNA-binding mutants (Figure 4D). It has been shown that DNA-bound STAT1 is protected from dephosphorylation and barred from nuclear exit [40], and we report here that the glutamyl mutants but not the wild-type protein resisted Tc45-catalyzed inactivation. These experiments collectively demonstrate that there must be a considerable amount of mutant phospho-STAT1 interacting with genomic DNA that does not participate in nucleocytoplasmic shuttling and resists inactivation by nuclear phosphatases.

### A low dissociation rate from DNA results in prolonged cytokine-induced nuclear accumulation

**Figure 5:**
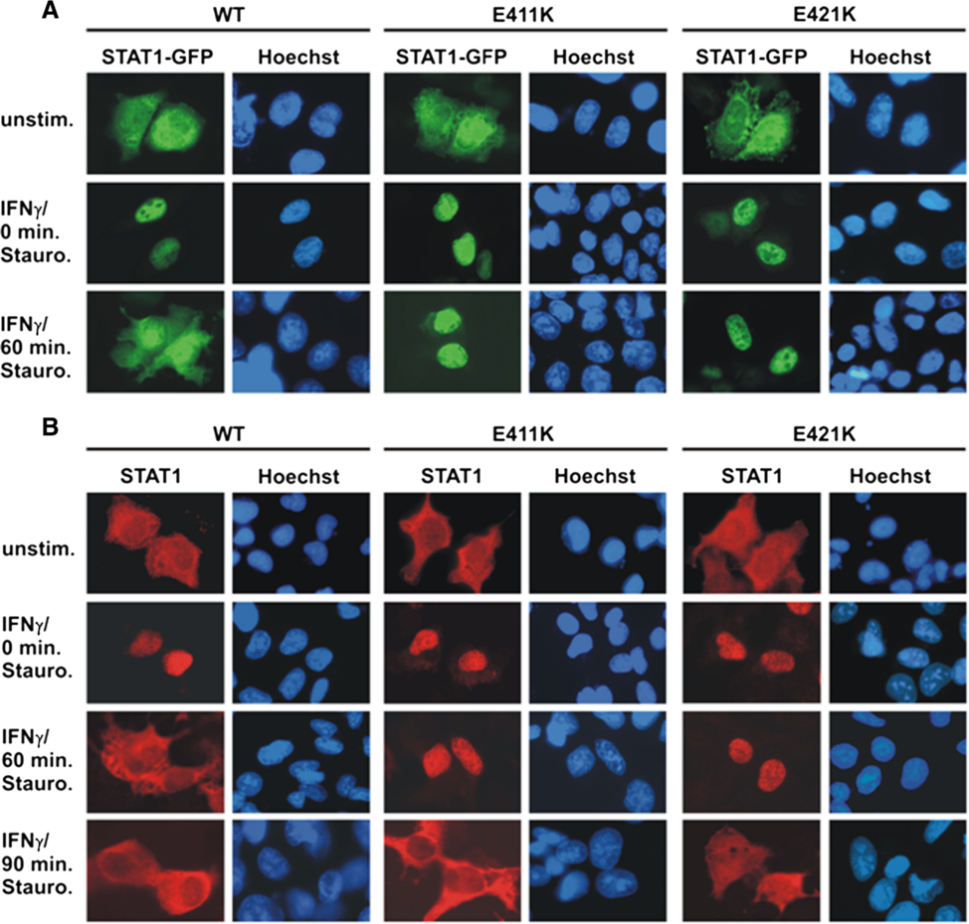
**STAT1 DNA-binding mutants with decreased dissociation from DNA exhibit a prolonged nuclear accumulation phase.** (**A**) Localization of GFP-tagged STAT1 variants in HeLa cells expressing either wild-type or mutant STAT1, in which glutamic acid residues at positions 411 and 421 were substituted for lysine. The cells were left unstimulated (top panel), stimulated with IFNγ for 45 min (middle panel), or pre-treated with IFNγ and subsequently exposed to 500 nM staurosporine for an additional 60 min (bottom panel). Fluorescence micrographs demonstrate the intracellular STAT1-GFP distribution before and after cytokine stimulation, as well as the effects of staurosporine treatment. Corresponding Hoechst-stained nuclei are shown. Note that, in the presence of the kinase blocker staurosporine, both glutamyl mutants remained in the nuclear compartment, whereas in cells expressing wild-type STAT1 the same treatment resulted in the collapse of STAT1 nuclear accumulation. (**B**) Time course of the collapse of nuclear accumulation in U3A cells expressing the indicated untagged STAT1 variants. Cells were either left untreated or treated for 45 min with IFNγ followed by subsequently exposure to 500 nM staurosporine for 0, 60 and 90 min, respectively. The nuclei of the methanol-fixed cells were stained with Hoechst dye and the samples processed for immunocytochemical detection of STAT1 using a pan-STAT1 antibody and appropriate Cy3-labeled secondary antibody.

The experiments presented thus far have shown that mutation of two critical glutamyl residues in the DNA-binding domain results in high-affinity DNA binding and defective tyrosine dephosphorylation of STAT1 upon stimulation of cells with IFNγ. Therefore, we wondered whether the resting distribution and the kinetics of cytokine-inducible nuclear accumulation differed between the mutant and wild-type STAT1 variants. For these experiments, we additionally mutated the glutamyl acid residues at positions 411 and 421 in positively charged lysyl residues and found that the resulting two novel point mutants closely mimicked the corresponding alanine mutant as described above (data not shown). The GFP fusion proteins of all three STAT1 variants (wild-type, E411K, and E421K, respectively) demonstrated a similar localization in resting HeLa cells, namely a pancellular distribution with a slightly elevated cytoplasmic concentration (Figure 5A). Replacement of the native glutamic acid residues at position 411 and 421 was without effect on the cytokine-induced nuclear accumulation, since the tyrosine-phosphorylated GFP fusions were imported normally into the nuclear compartment. However, when IFNγ−prestimulated cells were subsequently treated for 60 min with the kinase inhibitor staurosporine, a striking difference between the two point mutants and wild-type STAT1 was detected. In HeLa cells expressing wild-type STAT1, staurosporine caused a rapid collapse of nuclear accumulation, while nuclear localization of the glutamyl mutants persisted despite the presence of staurosporine. Thus, both point mutations significantly retarded the nuclear residence time of STAT1, but did not completely prevent the collapse of nuclear accumulation, since after 120 min of staurosporine exposure the former resting distribution of STAT1 was again achieved (data not shown). Thus, not surprisingly, insensitivity to pharmacological kinase inactivation resulted not only in elevated levels of tyrosine-phosphorylated STAT1 (Figures 1 and 2), but also in a markedly prolonged phase of nuclear accumulation. Additionally, we found that, in the absence of staurosporine, the nuclear retention time was considerably prolonged for the mutant STAT1 proteins during IFNγ−induced stimulation (data not shown).

To exclude the possibility that the differential nuclear accumulation kinetics seen for the glutamyl mutants is an artefact resulting from the presence of the GFP domain, we confirmed this finding by means of immunocytochemical staining in U3A cells expressing recombinant, untagged STAT1 (Figure 5B). Similarly to the GFP adducts expressed in HeLa cells, the respective glutamyl mutants showed an unaltered resting distribution and accumulated normally in the nuclei of IFNγ−stimulated U3A cells. However, after 60 min of staurosporine addition to the cells, the mutant STAT1 molecules were still predominantly localized in the nucleus, whereas the resting distribution of the wild-type protein had already been restored at that time point. Following 90 min of staurosporine exposure, the nuclear accumulation of both mutants had also collapsed, demonstrating that the DNA-binding mutants were less sensitive to kinase inhibition. This finding in U3A cells confirmed that the reduced dephosphorylation rate and prolonged nuclear accumulation are inherent properties of the glutamyl mutants, which result directly from their slow off-rate from DNA.

### High-affinity DNA binding crucially impairs transcriptional responses

**Figure 6:**
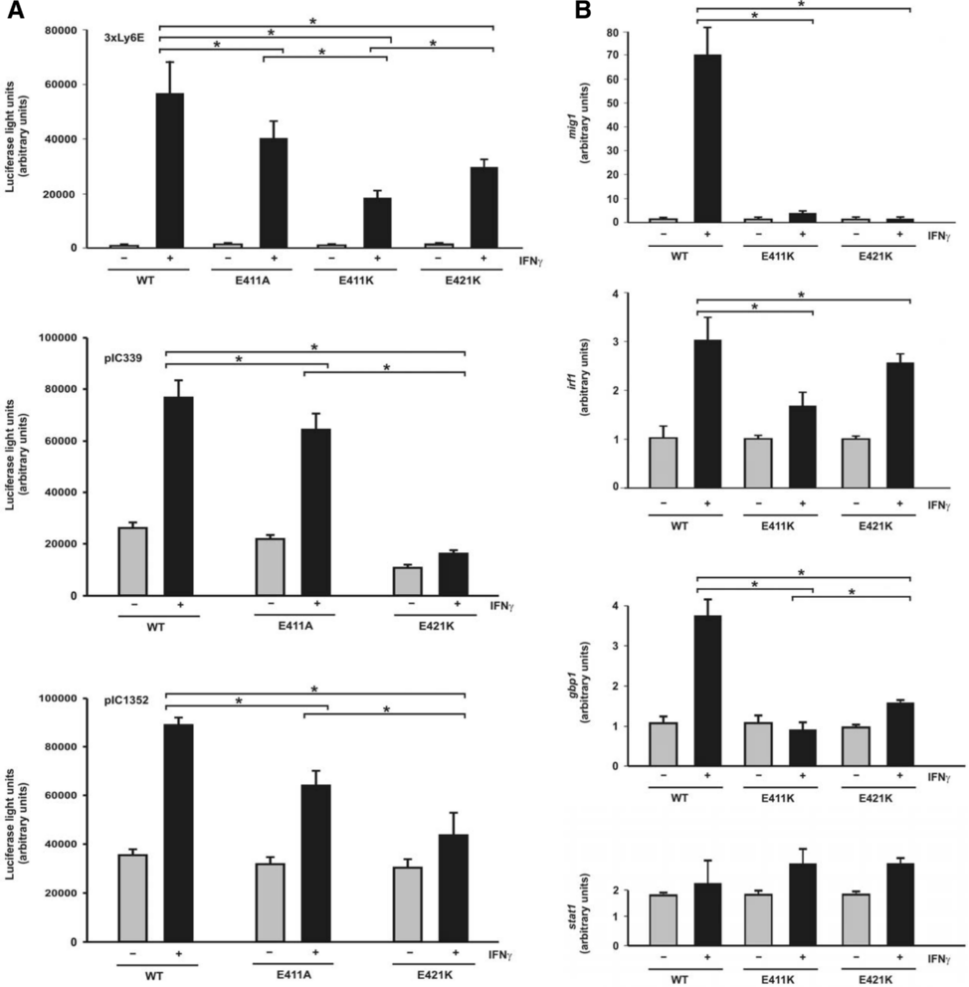
**Suppressed transcriptional responses of STAT1 mutants with high-affinity GAS binding.** (**A**) Decreased reporter gene activation in U3A cells expressing STAT1 DNA-binding mutants as compared to wild-type protein. U3A cells were transiently transfected with expression plasmids coding for the indicated STAT1 variants, a luciferase reporter construct and a constitutively expressed β-galactosidase gene used for normalization. The three reporter constructs used in these experiments contained either a triple GAS site from the Ly6E promoter (3xLy6E) or a 339 base pair or 1352 base pair fragment from the native ICAM-1 promoter (pIC339 and pIC1352). On the next day, the cells were either left untreated (gray columns) or stimulated for 6 hours with 5 ng/ml IFNγ (black columns) before, in whole cell extracts, luciferase luminescence and ß-galactosidase activity were measured. (**B**) Reduced activation of three endogenous STAT1 target genes by the E411K and E421K mutants. RT-PCR assays were performed with U3A cells expressing either STAT1 wild-type, E411K or E421K. Expression levels of the *irf1*, *mig1*, *gbp1*, and for control *stat1* gene before and after 6 hours stimulation with 5 ng/ml IFNγ (gray and black columns, respectively) are shown. Gene induction was normalized to the expression of the house-keeping gene *gapdh*. The data are presented as means and standard deviations from at least three independent experiments. Statistical significance between the groups of IFNγ−stimulated cells expressing the indicated STAT1 variants is marked by asterisks.

The impact of high-affinity DNA binding on gene transcription was next investigated. Reporter gene assays were performed to assess the consequence of a decreased dissociation rate from DNA on gene expression. Using a luciferase reporter with a synthetic promoter containing three strong GAS sites separated by 10 bp (3xLy6E), we found that all STAT1 variants tested displayed transcriptional responses upon stimulation of reconstituted U3A cells with IFNγ (Figure 6A). However, reporter gene induction was significantly repressed in cells expressing either of the glutamyl mutants as compared to the wild-type protein. STAT1-E411K displayed the lowest reporter gene activation of the mutants under investigation, demonstrating that the transcriptional activity decreased from wild-type > E411A > E411K (p = 0.049 and p < 0.001, respectively). Similar results were also obtained for STAT1-E421K using two reporters containing native fragments from the ICAM-1 promoter, termed pIC339 and pIC1352 (Figure 6A). Thus, exchange of the negatively charged glutamyl acid residue at position 411 for either a neutral or positively charged amino acid stepwise diminished the transcriptional response on a reporter gene with a strong cytokine-driven promoter.

We then used real-time RT-PCR assays to probe the induction of three endogenous IFNγ−responsive genes in transfected U3A cells. Again, the mutants failed to reach the transcriptional activity of the wild-type protein (Figure 6B). While constitutively expressed recombinant *stat1* mRNA was detected in all samples as expected (p > 0.05), there was a significant reduction in *irf1* mRNA synthesis in U3A cells expressing the E411K and E421K mutant as compared to wild-type STAT1 (p = 0.029 and p = 0.003, respectively). Induction of the *gbp1* and *mig1* gene was also critically impaired or even completely abolished by replacing either of the glutamyl residues (for all comparisons p < 0.03).

## Discussion

Previously, it was shown by *in vitro* studies using purified STAT1 that tyrosine-phosphorylated STAT1 dimers bound to DNA are protected from the inhibitory activity of nuclear phosphatases and barred from nuclear exit [40]. Although the physiological significance of this finding remains unclear, it has been suggested that a slow off-rate from genomic DNA critically compromises the STAT′s functions as potent transcription factors for a limited number of target genes. The corresponding DNA-binding mutants of STAT1 created so far display either a decreased affinity for DNA or a complete failure to discriminate between GAS and non-GAS elements. Thus, not surprisingly, both resulted in defective transcriptional activity [13,41]. However, the behavior of a hypothetical DNA-binding mutant with preserved GAS recognition and enhanced DNA-binding affinity surpassing that of the wild-type protein has not been studied so far. It is surmised that such a DNA-binding mutant would be a useful tool to dissect STAT1 signal transduction in terms of nucleocytoplasmic shuttling, cytokine-induced nuclear accumulation, and activation of target genes. As presented here, we have created such a point mutant which has allowed us to systematically investigate the transcriptional consequences of enhanced DNA binding. Using this mutant, we have revealed a simple molecular mechanism that enables STAT dimers to dissociate from non-target DNA in order to continue their search for GAS sites. We have demonstrated that a high off-rate from genomic DNA is required as a key feature for target gene finding, which allows for the efficient transmission of extracellular signals into transcriptional responses.

In an attempt to characterize the biological effects of increased protein-DNA interactions at a transcriptional level, we performed a mutational study on STAT1 and assessed the resulting mutants for their capability to bind sequence-specifically to DNA and activate interferon-responsive target genes. Most of the STAT1 point mutants generated with substitutions in the DNA-binding domain showed a reduced affinity for DNA and were, therefore, inappropriate to test the functional consequences of high-affinity DNA binding for gene expression. However, we identified two single point mutants that fulfilled our expectations for an enhanced binding to GAS sites. Replacement of two glutamic acid residues in the DNA-binding domain, although not interfering with the recognition of GAS elements, independently stabilizes preformed STAT1-DNA complexes. The presence of negatively charged residues at position 411 and 421 is required for the release of STAT1 dimers from DNA, as their substitution with either alanine or a positively charged lysyl residue remarkably reduced the dissociation rate from both GAS and GAS-like elements. The striking finding that enhanced GAS binding is associated with a dramatically reduced gene expression in cytokine-stimulated cells clearly underlines the significance of intact nucleocytoplasmic shuttling for full transcriptional activation. Moreover, it suggests that a limited residence time in the nucleus is an inherent property of STAT1 signal transduction and, conversely, a reduced dissociation rate from GAS elements results in suppressed gene induction.

Available crystallographic data have revealed that the glutamyl residue 411 does not directly contact specific nucleotide bases or the sugar-phosphodiester backbone of DNA, but in the DNA-bound form it has nevertheless free access to the DNA molecule, suggesting that there may be some minor structural flexibility within the STAT1 DNA-binding domain (see Figure 1A). It has been reported that residue 421 can accept hydrogen bonds from guanine in the minor groove, although the precise interface between the surface of the STAT1 DNA-binding domain and the DNA double helix in the proximity to E421 is not known due to the superimposition of non-equivalent base pairs at these positions [3]. The functional relevance of the two glutamyl residues can best be regarded as an off-switch to release STAT1 dimers from DNA, so that they become a readily accessible substrate for the inactivating nuclear phosphatase. The presence of a glutamic acid residue with a terminal carboxyl group adjacent to phosphate groups in the DNA backbone facilitates the fast disassembly of STAT1-DNA complexes possibly via electrostatic repulsion. Interestingly, these residues are directly engaged in the discrimination between canonical and non-canonical binding sites, since its replacement by alanine results in a mutant with preserved GAS recognition and a broadened spectrum of potential binding sites (see Figure 4C). This finding suggests that the repulsive effect on DNA binding exerted by these residues is independent of the underlying DNA sequences and occurs at classical GAS, GAS-like or even non-GAS sites. The native glutamyl residues seem to facilitate the release of STAT1 dimers from DNA via electrostatic interactions, thereby increasing the number of STAT1 molecules participating in productive nucleocytoplasmic shuttling.

In the wild-type molecule, the fast dissociation from DNA contributes to the coupling of DNA release and subsequent tyrosine dephosphorylation to transcriptional activation. Under conditions of cytokine stimulation the fast release from DNA ensures that the intracellular concentration of tyrosine-phosphorylated STAT1 is always limited due to the high tyrosine phosphatase activity in the nucleoplasma. In the DNA-binding mutants E411A/K and E421K, this coupling between the recruitment to genomic DNA and their fast dephosphorylation is critically disturbed, since these mutants are more than the wild-type protein stacked on genomic DNA in complexes, which may also contain co-expressed native STAT1 (see Figures 1B and 2A,B). Due to the reduced number of cycling STAT1 dimers (Figure 3), their cytokine-induced transcriptional response is substantially limited (Figure 6). The prolonged nuclear residence time of the glutamyl mutants following cytokine stimulation of cells (Figure 5) appears to directly reflect their decreased tyrosine dephosphorylation (Figure 4D), suggesting that they are retained in a DNA-bound state at transcriptionally inert genomic loci. Tyrosine-phosphorylated native STAT1 molecules form heterodimers with the co-expressed recombinant STAT1 mutants as detected by gelshift experiments (Figure 2B), which are integrated into DNA-bound STAT complexes and protected from fast inactivation (Figure 2A). Thus, paradoxically, despite their increased GAS binding and elevated concentration in the nuclear compartment, where transcription exclusively takes place, the mutants are nevertheless weaker transcriptional activators.

Interestingly, by introducing a neutral or a positively charged functional group at position 411, we generated a graduated series of STAT1 variants (glutamate > alanine > lysine) with stepwise diminished transcriptional activity at an artificial reporter gene construct. Thus, changing the electric charge of this residue permits interference with gene induction simply by shifting the amount of STAT1 dimers to a DNA-bound state in which they are prevented from freely shuttling between cytoplasm and nucleus. From our experiments, we cannot conclude whether the impaired transcriptional activity at native target genes detected for the mutants results from a diminished exchange rate at a single promoter or merely reflects decreased promoter occupancy due to predominant deposition at low-affinity DNA-binding sites. However, we observed that cytokine stimulation leads to high nuclear concentrations of mutant STAT1, which clearly exceed that of the wild-type protein (see Figure 3A). This finding suggests that mutant STAT1 preferentially deposits outside transcriptionally active sites. In this scenario, a limited number of high-affinity GAS sites compete with the virtually unlimited amount of non-GAS sequences within the entire genome for binding to STAT1. In interferon-stimulated cells, phospho-STAT dimers retained in the nucleus may not be exclusively bound to GAS sites, but are additionally recruited to an overwhelming reservoir of unspecific, low-affinity DNA-binding sites, from which they are released with very high exchange rates [42]. Interestingly, Lerner and colleagues had previously shown that STAT3 and glucocorticoid receptor assembled at the α2-macroglobulin promoter into an enhanceosome for which continued renewal of both transcription factors was required for full transcriptional activity [43].

## Conclusions

In summary, we present evidence showing that the presence of two single glutamic acid residues in the DNA-binding domain adjacent to the DNA backbone sequence-independently weakens the binding to DNA and is required for full transcriptional activation of cytokine-driven target genes. The high dissociation rate from non-GAS sites ensures that tyrosine-phosphorylated STAT1 dimers can successfully scan genomic DNA for the presence of specific GAS sites, at which they assemble into transcriptional active complexes until they are finally dephosphorylated for nuclear exit. Furthermore, we demonstrate that not a high affinity for GAS sites, but rather the inherent difference in the off-rates between specific and non-specific binding sites crucially determines the function of STAT proteins as transcriptional regulators.

## Methods

### Cell culture

HeLa cells were cultured at 37^ο^C in a humidified 5% CO_2_ atmosphere in Quantum 101 medium (PAA Laboratories) supplemented with 5% fetal calf serum (FCS; Biochrom), 1% penicillin, and 1% streptomycin. STAT1-negative U3A cells, originally derived by Müller and colleagues [44], were cultured in Dulbecco’s modified Eagle’s medium supplemented with 10% FCS, 1% penicillin, 1% streptomycin, and 0.04 μg/ml puromycin. Transfection was achieved with Lipofectamine plus (Invitrogen) according to the manufacturer's recommendation. Twenty-four hours after transfection, cells were either left unstimulated or stimulated with 5 ng/ml human IFNγ (Biomol). Subsequently, cells were incubated with 500 nM staurosporine (Sigma) for the time periods indicated.

### Plasmids

The plasmid pEGFP-N1-STAT1, which coded for full-length human STAT1 (amino acids 1–746) fused carboxy-terminally to green fluorescent protein (GFP), has been described [45]. For the detection of untagged protein, STAT1 cDNA was cloned in the expression vector pcDNA3.1 (Invitrogen). The plasmid pSTAT1-NES-GFP contained a transferable nuclear export signal (NES) activity (amino acids 367–427 of STAT1) situated between the cDNAs for full-length STAT1 and GFP, as described [39]. Mutations in each of these expression vectors were introduced by site-directed point mutagenesis using the QuikChange kit from Stratagene, as recommended by the manufacturer. The native glutamic acid codons at position 411 and 421 were substituted for either alanine or lysine. All mutations were verified by standard didesoxy termination DNA sequencing (Seqlab).

### Fluorescence microscopy

For direct fluorescence microscopic localization of GFP-tagged STAT1, transiently transfected cells were treated as described and subsequently fixed in 3.7% paraformaldehyde in phosphate-buffered saline (PBS) for 15 min at room temperature (RT). Subsequently, nuclei were stained for 10 min with 5 μg/ml Hoechst 33258 (Sigma) and the samples were mounted in fluorescence mounting medium (Southern Biotech). Fluorescence microscopy was performed using a Leica DM5000B microscope equipped with appropriate fluorescence filters. Images were obtained with a CCD camera and further processed with the Leica QWin software.

### Immunocytochemistry

Immunocytochemical detection of untagged STAT1 was carried out in U3A cells expressing either wild-type or mutant STAT1. Adherent cells grown on chamber slides were either left untreated or treated with IFNγ for 45 min. Interferon-stimulated cells were additionally incubated in the presence of 500 nM staurosporine for an additional 0, 60 or 90 min and then fixed with methanol for 20 min at −20°C. After two washes in PBS, the cells were permeabilized with 1.0% Triton X-100 in PBS and non-specific binding was blocked by incubation with 25% FCS/PBS for 45 min at RT. The samples were then incubated for 45 min with anti-STAT1 antibody C-24 (Santa Cruz) diluted 1:1000 in 25% FCS/PBS. After three washes in PBS they were incubated with Cy3-conjugated secondary antibody (Dianova), diluted 1:500 in PBS, for 45 min at RT followed by nuclear staining with Hoechst dye. Finally, the samples were mounted and images were captured by fluorescence microscopy.

### Digitonization

Adherent HeLa cells expressing GFP-fusion proteins of STAT1 were stimulated for 45 min with IFNγ to induce nuclear accumulation of STAT1. Then cells were either left untreated or permeabilized in the presence of 50 μg/ml digitonin (Roche) in transport buffer (0.2% Triton X-100, 10 mM KCl, 1.5 mM MgCl_2_, 10 mM Hepes, pH 7.4, 1 mM DTT, Complete protease inhibitors from Roche) for 6 min on ice. After two washes in transport buffer, cells were fixed for 15 min at RT with 3.7% paraformaldehyde in PBS followed by staining with Hoechst dye. The presence of STAT1-GFP in the nuclei was probed by means of direct fluorescence microscopy.

### *In vitro* dephosphorylation assay

For *in vitro* dephoshorylation assays, 10 μl of cytosolic extracts from U3A cells expressing mutant STAT1 proteins were mixed with a similar volume of dephosphorylation buffer containing 25 mM Tris–HCl, pH 7.5, 50 mM KCl, 5 mM EDTA, and 0.3 mg/ml bovine serum albumin. Then DTT was added to a final concentration of 2 mM before the samples were incubated at 30°C with 2 U of the T-cell protein tyrosine phosphatase Tc45 (Biomol International, Plymouth, USA) for 0 min, 15 min and 30 min, respectively. Dephosphorylation reactions were stopped by adding SDS sample buffer and boiling the samples for 3 min. The amount of tyrosine-phosphorylated STAT1 in each sample was tested by means of Western blotting.

### Western blotting

Cells grown on 6-well dishes were lysed in 30 μl cytoplasmic extraction buffer (0.2% Nonidet P40, 10 mM KCl, 1 mM EDTA, 10% glycerol, 20 mM Hepes, pH 7.4, 50 mM NaF, 1 mM vanadate, 1 mM DTT, 0.1 mM PMSF, Complete protease inhibitors) for 5 min on ice. The lysates were spun at 16000 g for 10 sec at 4°C. The supernatants were recentrifuged at 16000 g for 5 min and the pellets resuspended in 30 μl nuclear extraction buffer (420 mM KCl, 1 mM EDTA, 20% glycerol, 20 mM Hepes, pH 7.4, 50 mM NaF, 1 mM vanadate, 1 mM DTT, 0.1 mM PMSF, Complete protease inhibitors) for 30 min on ice and spun for 15 min at 16000 g. The isolated or combined cytoplasmic and nuclear extraction lysates were boiled in SDS sample buffer. Proteins were then resolved by 10% SDS-PAGE and subsequently transferred to nitrocellulose membranes. The membranes were incubated with a polyclonal antibody specific for phospho-STAT1-Tyr^701^ (Cell Signaling) and then with a horseradish peroxidase-conjugated secondary antibody (Dako). Bound immunoreactivity was detected using the enhanced chemiluminescence reaction (Pierce). Subsequently, the blots were stripped for 60 min at 60°C in 2% SDS, 0.7% β-mercaptoethanol, and 62.5 mM Tris–HCl, pH 6.8. Finally, the blots were reprobed with the polyclonal pan-STAT1 antibody C-24 followed by incubation with secondary antibodies. The efficiency of nuclear/cytoplasmic fractionation was assessed by simultaneously incubating blot membranes with rabbit lamin A (H-102, Santa Cruz) and mouse β-tubulin antibodies (clone TUB2.1, Sigma) followed by detection with secondary IRDye 680LT and 800CW antibodies visualized on a LI-COR Odyssey imaging machine.

### Electrophoretic mobility shift assay (EMSA)

HeLa or U3A cells were transiently transfected with pSTAT1-GFP or pcDNA3.1-STAT1 coding for either wild-type or mutant STAT1. The cells were allowed to recover for 24 hours and then either left unstimulated or stimulated for 45 min with 5 ng/ml of IFNγ followed by staurosporine treatment. Cell extracts were prepared as described above. To prevent dephosphorylation and proteolysis, all cell extracts contained a protease inhibitor cocktail (Roche), 1 mM vanadate, and 10 mM NaF. Four microliters of each extract were incubated with 1 ng ^32^P]-labeled duplex oligonucleotide probes, generated by an end-filling reaction using Klenow fragment (New England Biolabs). The following duplex oligonucleotides were used (4 bp overhangs at the 5^′^ ends are not included; the respective antisense oligos are not listed; GAS sites are underlined): M67, 5^′^-CGACATTTCCCGTAAATCTG-^′^3; 2x GAS, 5^′^-CGTTTCCCCGAAATTGACGGATTTCCCCGAAAC-^′^3; GAS-nonGAS, 5^′^-CGTTTCCCCGAAATTGACGGATTTACCCCAAC-^′^3; and 2x nonGAS, 5^′^-CGTTTACCCCAAATTGACGGATTTACCCCAAC-^′^3. For competition experiments, the EMSA reactions were equilibrated for 15 min at RT before incubation with a 750-fold molar excess of unlabeled M67 DNA for the indicated times. In supershift assays, 20 ng of the STAT1-specific antibody C-24 were preincubated with the shift reaction for 15 min at RT. The reactions were loaded on a 4% 29:1 acrylamide:bisacrylamide gel at 4°C, as described [45]. STAT1 DNA-binding activity was visualized with a phosphoimaging system (BAS-1000, Fujifilm) using the computer programs BAS reader and TINA version 2.08.

### Reporter gene assay

U3A cells grown on 48-well plates were transiently transfected with the following amounts of cDNAs added into a single well: 250 ng of the respective STAT1 expression plasmid, 70 ng of a β-galactosidase reporter plasmid (Stratagene), and 200 ng of an IFNγ−sensitive reporter construct. Luciferase reporters contained either a triple Ly6E STAT-binding site (termed 3xLy6E) or the 5^′^-region of the human intercellular adhesion molecule 1 (ICAM-1) gene 339 bp and 1352 bp relative to the transcription start site (termed pIC339 and pIC1352, respectively) [46,47]. Twenty-four hours post-transfection, cells were either left unstimulated or treated for 6 hours with IFNγ. Whole cell extracts were prepared and measured for luciferase (Promega) and β-galactosidase activities. The data were normalized for the expression of β-galactosidase. For each STAT1 variant and stimulation mode, six independent samples were tested and the experiment was repeated at least three times. Differences in gene activation between IFNγ−stimulated cells expressing the indicated STAT1 variants were assessed using Student’s *t* tests and Mann-Whitney-Wilcoxon tests, where appropriate. Statistical significance was defined as p < 0.05.

### Real-time PCR

The transcriptional activities of wild-type and mutant STAT1 were assessed by means of real-time PCR. Gene-specific primers for three endogenous transcripts (*irf1*, *mig1*, and *gbp1*) as well as for *stat1* and *gapdh* were designed using Primer 3 software (Applied Biosystems) in order to amplify fragments of about 200 bp in length. The following primer pairs were used: IRF1F, 5^′^-AGCTCAGCTGTGCGAGTGTA-^′^3; IRF1R, 5^′^-TAGCTGCTGTGGTCATCAGG-^′^3; MIG1F, 5^′^-CCACCGAGATCCTTATCGAA-^′^3; MIG1R, 5^′^-CTAACCGACTTGGCTGCTTC-^′^3; GBP1F, 5^′^-GGTCCAGTTGCTGAAAGAGC-^′^3; GBP1R, 5^′^-TGACAGGAAGGCTCTGGTCT-^′^3; GAPDHF, 5^′^-GAAGGTGAAGGTCGGAGTC-^′^3; GAPDHR, 5^′^-GAAGATGGTGATGGGATTTC-^′^3; STAT1F, 5^′^-CCGTTTTCATGACCTCCTGT-^′^3; and STAT1R, 5^′^-TGAATATTCCCCGACTGAGC-^′^3. Twenty-four hours after transfection, U3A cells were incubated for an additional 15 hours with 1% FCS diluted in Dulbecco’s modified Eagle’s medium. The cells were then either left untreated or stimulated for 6 hours with IFNγ. RNA was isolated with the peqGold Total RNA kit (Peqlab) and first-strand cDNA synthesis was performed using the Superscript III kit from Invitrogen. The real-time PCR reactions were carried out in a total volume of 25 μl, containing 25 ng cDNA, 70 nM of each specific primer pair, and 12.5 μl SYBR Green (Thermo Fisher Scientific). The following protocol was applied: a denaturation step at 95°C for 15 min, and 45 cycles of denaturation at 95°C for 15 s, annealing at 55°C for 30 s, and extension at 72°C for 30 s. Following the final amplification, a melting curve analysis was run via a temperature gradient from 60°C to 95°C in 0.5°C increment steps, fluorescence being measured at each temperature for a period of 10 s. All reactions were carried out in at least triplicate for each sample. The relative expression of a transcript was normalized to the expression of *gapdh* as determined for each sample. Using the Realplex 1.5 software from Eppendorf, the threshold (Ct) at which the cycle numbers were measured was adjusted to areas of exponential amplification of the traces. The ΔΔCt-method was used to determine comparative relative expression levels, the formula 2^-(ΔCt target - ΔCt reference sample)^ being applied as described previously [48]. Statistical analysis was as described above.

## Abbreviations

DTT: dithiothreitol; EMSA: electrophoretic mobility shift assay; GAS: gamma-activated site; GFP: green fluorescent protein; IFNγ: interferon-γ; JAK: Janus-activated kinase; NES: nuclear export signal; SDS-PAGE: sodium dodecyl sulfate-polyacrylamide gel electrophoresis; PCR: polymerase chain reaction; RT-PCR: reverse-transcriptase PCR; SH2 domain: Src homology 2 domain; STAT1: signal transducer and activator of transcription 1.

## Competing interests

The authors declare that they have no competing interests.

## Authors’ contributions

VK carried out the majority of the experiments, designed or contributed to the design of the experiments and to the preparation of the manuscript. JS contributed to the final preparation of the manuscript and to the analysis of gene expression data. VR participated in the discussion concerning the characterization of STAT1 DNA binding. TM designed and supervised the experiments and drafted the manuscript. All authors read and approved the final manuscript.

## Acknowledgements

The authors gratefully acknowledge the excellent technical assistance of Miriam Gehring and Sandra Sieber from the University of Marburg. We kindly thank Dr. Uwe Vinkemeier, University of Nottingham, for valuable reagents and discussions. The research on this subject was funded by a grant from the Deutsche Forschungsgemeinschaft to TM.
